# Cerebral inflammation and mobilization of the peripheral immune system following global hypoxia-ischemia in preterm sheep

**DOI:** 10.1186/1742-2094-10-13

**Published:** 2013-01-24

**Authors:** Reint K Jellema, Valéria Lima Passos, Alex Zwanenburg, Daan RMG Ophelders, Stephanie De Munter, Joris Vanderlocht, Wilfred TV Germeraad, Elke Kuypers, Jennifer JP Collins, Jack PM Cleutjens, Ward Jennekens, Antonio WD Gavilanes, Matthias Seehase, Hans J Vles, Harry Steinbusch, Peter Andriessen, Tim GAM Wolfs, Boris W Kramer

**Affiliations:** 1 School of Mental Health and Neuroscience, Maastricht University, Universiteitssingel 40, Maastricht, 6229 ER, The Netherlands; 2 Department of Pediatrics, Maastricht University Medical Center, PO box 5800, Maastricht, 6202 AZ, The Netherlands; 3 Department of Methodology and Statistics, Maastricht University, P. Debyeplein 1, Maastricht, 6229 HA, The Netherlands; 4 Department of Biomedical Technology, Maastricht University, Universiteitssingel 50, Maastricht, 6229 ER, The Netherlands; 5 Department of Transplantation Immunology, Tissue Typing Laboratory, Maastricht University Medical Center, PO box 5800, Maastricht, 6202 AZ, The Netherlands; 6 Department of Internal Medicine, Division of Haematology, Maastricht University, Universiteitssingel 50, Maastricht, 6229 ER, The Netherlands; 7 Department of Pathology, Maastricht University Medical Center, PO box 5800, Maastricht, 6202 AZ, The Netherlands; 8 Department of Child Neurology, Maastricht University Medical Center, PO box 5800, Maastricht, 6202 AZ, The Netherlands; 9 School of Oncology and Developmental Biology, Maastricht University, Universiteitssingel 50, Maastricht, 6229 ER, The Netherlands; 10 Neonatal Intensive Care Unit, Maxima Medical Centre, De Run 4600, Veldhoven, 5504 DB, The Netherlands; 11 Department of Clinical Physics, Maxima Medical Centre, De Run 4600, Veldhoven, 5504 DB, The Netherlands

**Keywords:** Hypoxic-ischemic encephalopathy, Inflammation, Microglia, Neutrophils, Oligodendrocytes, Preterm, Spleen

## Abstract

**Background:**

Hypoxic-ischemic encephalopathy (HIE) is one of the most important causes of brain injury in preterm infants. Preterm HIE is predominantly caused by global hypoxia-ischemia (HI). In contrast, focal ischemia is most common in the adult brain and known to result in cerebral inflammation and activation of the peripheral immune system. These inflammatory responses are considered to play an important role in the adverse outcomes following brain ischemia. In this study, we hypothesize that cerebral and peripheral immune activation is also involved in preterm brain injury after global HI.

**Methods:**

Preterm instrumented fetal sheep were exposed to 25 minutes of umbilical cord occlusion (UCO) (n = 8) at 0.7 gestation. Sham-treated animals (n = 8) were used as a control group. Brain sections were stained for ionized calcium binding adaptor molecule 1 (IBA-1) to investigate microglial proliferation and activation. The peripheral immune system was studied by assessment of circulating white blood cell counts, cellular changes of the spleen and influx of peripheral immune cells (MPO-positive neutrophils) into the brain. Pre-oligodendrocytes (preOLs) and myelin basic protein (MBP) were detected to determine white matter injury. Electro-encephalography (EEG) was recorded to assess functional impairment by interburst interval (IBI) length analysis.

**Results:**

Global HI resulted in profound activation and proliferation of microglia in the hippocampus, periventricular and subcortical white matter. In addition, non-preferential mobilization of white blood cells into the circulation was observed within 1 day after global HI and a significant influx of neutrophils into the brain was detected 7 days after the global HI insult. Furthermore, global HI resulted in marked involution of the spleen, which could not be explained by increased splenic apoptosis. In concordance with cerebral inflammation, global HI induced severe brain atrophy, region-specific preOL vulnerability, hypomyelination and persistent suppressed brain function.

**Conclusions:**

Our data provided evidence that global HI in preterm ovine fetuses resulted in profound cerebral inflammation and mobilization of the peripheral innate immune system. These inflammatory responses were paralleled by marked injury and functional loss of the preterm brain. Further understanding of the interplay between preterm brain inflammation and activation of the peripheral immune system following global HI will contribute to the development of future therapeutic interventions in preterm HIE.

## Background

Hypoxic-ischemic encephalopathy (HIE) is one of the most important causes of brain injury in preterm infants [1]. Preterm infants suffering from HIE develop cognitive disorders in 25 to 50% of all cases and 5 to10% suffer from severe motor deficits (such as cerebral palsy) [2]. The hippocampus plays a key role in cognition and several studies suggest that hypoxia-ischemia (HI)-induced injury to the hippocampus may predispose to cognitive disorders later in life [3-7]. Motor deficits in preterm HIE are mainly attributable to injury of white matter in the immature brain [1]. Despite the high prevalence of neurological sequelae, no therapeutic interventions are available to treat HIE in preterm infants. Cooling therapy, which has been shown to improve neurodevelopmental outcome in mild cases of HIE in term infants, is associated with adverse outcomes in preterm infants and has therefore not yet been established as standard clinical care for this vulnerable patient group [8-10].

White matter injury, the clinical hallmark of preterm HIE, is caused by injury to the highly vulnerable immature oligodendrocytes in the preterm brain. HI-induced damage to immature oligodendrocytes impedes effective differentiation into mature myelinating oligodendrocytes leading to hypomyelination of the preterm brain [11-13]. Microglial activation is considered to be involved in the injury to immature oligodendrocytes [13]. Microglia are the resident innate immune cells in the brain and play a central role in the initiation of an inflammatory response aimed at resolving injury caused by HI [14-16]. Excessive activation of microglia, however, results in a detrimental cerebral inflammatory response with neurotoxic consequences [15,16]. In addition to cerebral inflammation, experimental data from adult rodent models of focal HI (stroke) suggest a role for the peripheral immune system in the etiology of cerebral HI. More precisely, several studies showed that acute brain injury after focal ischemia is followed by a massive activation of the peripheral immune system with rapid mobilization of immune effector cells from the spleen [17,18]. These mobilized effector cells can invade the brain and aggravate the existing injury [17].

Given the importance of cerebral inflammation and peripheral immune system activation in focal HI of the adult brain, we hypothesized that similar inflammatory responses are involved in the etiology of preterm brain injury following global HI. To test this hypothesis, preterm instrumented sheep were exposed to 25 minutes of umbilical cord occlusion (UCO) at 0.7 gestation. At this time of gestation, neurodevelopment of fetal sheep is equivalent to that of a preterm human infant of 28 to 32 weeks [19-21]. During this neurodevelopmental stage of the human and ovine fetus, the preterm brain is highly prone to develop white matter injury following global HI [11-13].

## Methods

### Animal experiments

The study was approved by the Animal Ethics Research Committee of Maastricht University, The Netherlands. Fetuses of time-mated Texel ewes were instrumented at 101 ± 1.1 (mean ± SD) days gestation. Before surgery, ewes received i.v. prophylactic antibiotics (1000 mg amoxicillin and 200 mg clavulanic acid). Anesthesia was induced by i.v. thiopenthal (15 mg/kg). After intubation, general anesthesia was maintained with 1 to 2% isoflurane guided by depth of sedation and supplemented by remifentanyl i.v. (0.75 μg/kg/min) for analgesia. Vital parameters and depth of sedation were continuously monitored by certified personnel. A catheter was placed in the maternal long saphenous vein to provide access for a peri-operative saline drip (250 mL/hour) and post-operative blood sampling and administration of the prophylactic antibiotics during four days.

Fetuses were catheterized with 3.5 French polyurethane umbilical vessel catheters (Tyco Healthcare Group, Mansfield, Massachusetts, USA) placed in the femoral artery and the brachial vein. Three custom-made electrocardiogram (ECG) shielded electrodes (Cooner Wire Co., Chatsworth, CA, USA) with silver plates (5 mm) were sewn on the chest for fetal heart rate recordings. Two pairs of custom-made electroencephalogram (EEG) shielded electrodes (Cooner Wire Co.) with silver tips were placed bilaterally on the dura over the parasagittal parietal cortex (5 mm and 15 mm anterior to point bregma and 10 mm lateral), with a subcutaneous silver reference electrode (10 mm) placed in the neck. The EEG electrodes were secured with cyanoacrylate glue and covered with fetal skin. All animals were instrumented with an inflatable vascular occluder (OC16HD, 16 mm, In Vivo Metric, Healdsburg, California, USA) placed around the umbilical cord. A catheter for amniotic pressure recording was placed in the amniotic sac. Before closure of the uterus 80 mg of Gentamycin was administered into the amniotic sac. All fetal catheters and leads were exteriorized through a trocar hole in the flank of the ewe.

After surgery, ewes were housed in a confined space to allow handling and continuous perfusion of the catheters with heparinized saline (25 IU/mL, 0.2 mL/hr). Surgical wounds were inspected daily and treated with chlortetracycline spray to prevent infection. Animals had *ad libitum* access to water and food. The welfare of the animals was monitored daily by certified personnel.

### Experimental design

**Figure 1:**
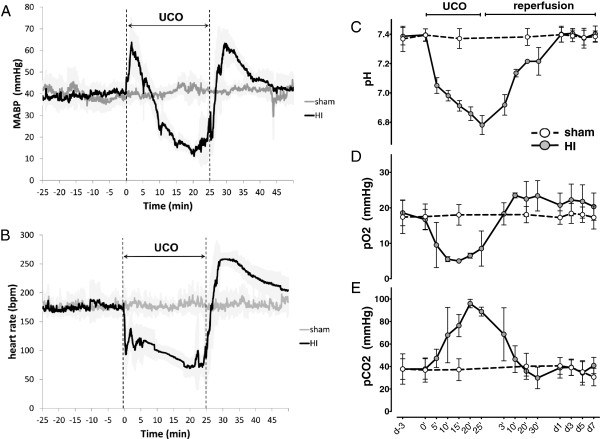
**Vital parameters and blood gases of sham and hypoxia-ischemia (HI) animals during umbilical cord occlusion (UCO).** (**A**) Fetal mean arterial blood pressure (MABP), the small deflections (every five minutes) in the MABP curve are caused by arterial blood gas sampling; (**B**) fetal heart rate (HR) in beats per minute (bpm); (**C**) blood gas: arterial pH; (**D**) blood gas: arterial partial oxygen pressure (pO_2_); (**E**) blood gas: arterial partial carbon dioxide pressure (pCO_2_). Shaded areas (MABP and HR) and error bars (blood gases) depict standard deviation (SD). Min/‘ = minutes, d = day.

Fetuses were instrumented at 101 ± 1 (mean ± SD) days of gestation (experimental day −4). After surgery, the ewe and her fetus were allowed to recover for four days. On experimental day 0, fetuses were randomly allocated to either be subjected to 25 minutes of umbilical cord occlusion (HI group, n = 8) or sham occlusion (sham group, n = 8). In the HI group, the occluder was rapidly inflated with sterile saline and complete occlusion was confirmed with a sudden drop in heart rate and subsequent arterial blood gas analysis indicating acidemia, hypoxia and hypercapnia (Figure 1). Such an insult has been previously shown to result in global HI and subsequent cerebral hypoperfusion [19,22]. After (sham) umbilical cord occlusion, a reperfusion period of 7 days followed. At the end of the experiment (experimental day 7), both ewe and fetus were euthanized by administration of pentobarbital (200 mg/kg).

### Data acquisition

Blood pressure, amniotic pressure, EEG and ECG data were acquired and digitized by a custom-made MPAQ unit (Maastricht-Programmable AcQuisition system, Maastricht Instruments BV, Maastricht, The Netherlands) with IDEEQ software (Maastricht Instruments BV). All data were sampled at 1000 Hz and stored on hard-disk for offline analysis. Analog filtering was applied to the ECG data, with a 1 Hz high-pass filter and a 200 Hz low-pass filter. Heart rate (beats per minute) was extracted from the ECG by R-top identification. Blood pressure and amniotic pressure data were not filtered. Fetal mean arterial blood pressure was calculated by online subtraction of the amniotic fluid pressure from the femoral artery pressure.

The EEG data were filtered using a 0.5 to 30 Hz 4^th^ order Butterworth band-pass filter. EEG signal with an amplitude >1000 μV was considered an artifact and removed from analysis (<1% of data). After filtering, EEG background analysis was performed using an amplitude- and time-threshold based algorithm [23]. Burst activity was defined as an epoch with an amplitude >30 μV and a duration >1 s in both channels. Interburst intervals (IBI) were defined as epochs with an amplitude <30 μV and a duration >3 s in both channels. Segments not meeting above criteria were classified as undefined. Using these criteria, mean IBI length per 30 minutes and per 24 hours segments was calculated and used as a surrogate for functional brain suppression for all animals during the period starting two days before UCO (day −2) until the end of the experiment (day 7).

### Immunohistochemistry brain

The fetal brain was removed from the skull and weighed. The right hemisphere was submersion fixated in ice-cold 4% paraformaldehyde for 3 months. Brain tissue was embedded in gelatin and serial coronal sections (50 μm) were cut on a Leica VT 1200S vibrating microtome (Leica Biosystems, Nussloch, Germany). Free floating sections at the level of mid-thalamus and posterior hippocampus were stained with a rabbit anti-ionized calcium binding adaptor molecule 1 (IBA-1) antibody (Wako Pure Chemical Industries, Osaka, Japan), a highly specific marker for microglia, to localize resting and activated microglia [24-26]. A mouse anti-O4 antibody (Merck Millipore, Billerica, MA, USA) was used to detect late oligodendrocyte progenitors and immature oligodendrocytes (hereafter collectively referred to as pre-oligodendrocytes; preOLs) and a rat anti-myelin basic protein (MBP) antibody (Merck Millipore) was used to detect myelin sheaths and myelin producing (mature) oligodendrocytes. A rabbit anti-myeloperoxidase (MPO) antibody (DAKO A0398, DAKO, Glostrup, Denmark) was used to detect neutrophils.

Endogenous peroxidase-activity was blocked by incubation with 0.3% H_2_O_2_ in Tris buffered saline (TBS, pH 7.4). Free floating sections were incubated overnight (anti-IBA-1, MBP and MPO) or during three days (anti-O4) at 4°C with the diluted primary antibody (1:1000 anti-IBA-1, 1:400 anti-O4, 1:2000 MBP and 1:1000 MPO) followed by incubation with a secondary donkey-anti-rabbit (anti-IBA-1 and MPO), donkey-anti-rat (MBP) or donkey-anti-mouse (anti-O4) biotin labeled antibody. The immunostaining was enhanced with Vectastain ABC peroxidase Elite kit (PK-6200, Vector Laboratories, Burlingame, CA, USA) followed by a nickel sulfate-diaminobenzidine (NiDAB) staining. Sections were mounted on gelatin-coated glass slides, air-dried, dehydrated in ascending ethanol concentrations and coverslipped with PerTex.

### Brain immunohistochemistry analysis

For the analysis of IBA-1 immunoreactivity (IR), digital images of the hippocampus, subcortical white matter (SCWM) and periventricular white matter (PVWM) were acquired at 100x magnification using an Olympus BX51 microscope (Olympus, Tokyo, Japan). In the regions of interest (ROIs) areal fraction of IBA-1 IR was determined with a standard threshold to determine positive staining using Leica Qwin Pro V 3.5.1 software (Leica, Rijswijk, The Netherlands). Within the hippocampus IBA-1 IR was additionally analyzed in the CA1-2, CA3 and dentate gyrus (DG) sub regions. IBA-1 IR areal fraction in the ROIs was assessed in six consecutive coronal sections (posterior hippocampus/mid-thalamus level) per animal (sham, n = 6; HI, n = 6) by an independent observer who was blinded to the experimental conditions.

**Figure 2:**
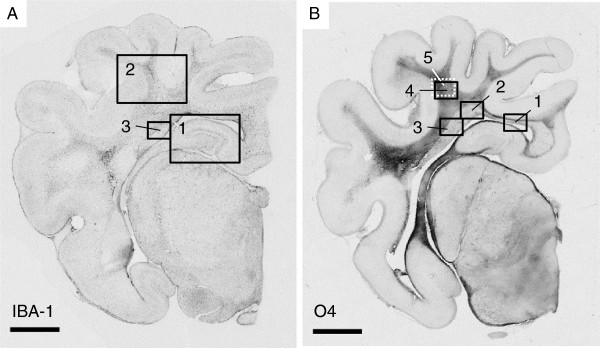
**Overview of regions of interest in the right hemisphere at the posterior hippocampus/mid-thalamus level.** (**A**) Regions of interest for the detection of IBA-1 immunoreactivity, 1 = hippocampus, 2 = subcortical white matter and 3 = periventricular white matter; (**B**) regions of interest for the detection of O4-positive cell density, 1 = medial periventricular white matter, 2 = intermediate periventricular white matter, 3 = lateral periventricular white matter, and 4 = subcortical white matter. Scale bar = 4 mm. The white dashed box (5) indicates in which region the myelin binding protein (MBP) images in Figure 7 were obtained.

Analysis of the O4 immunohistochemical staining clearly showed that within the periventricular white matter three sub-regions had region-specific preOL characteristics in sham animals that responded differently to global HI. Therefore O4 staining was assessed in these three different regions of interest in the PVWM. In addition, O4 staining was assessed in the SCWM. Regions of interest are indicated in Figure 2.

To assess O4 immunoreactivity, we adapted the method previously reported by Back *et al.*[11]. A differential count was performed, discriminating between immature (ring-shaped membrane staining, no processes), mature (ring-shaped membrane staining, extensively branched processes) and degenerative (fragmented membrane staining, fragmentation of processes, signs of cell death; nuclear condensation and apoptotic bodies) phenotype of the O4 positive cells. The sum of the differential count resulted in the total number of O4 positive cells. Differential counts were performed in six consecutive coronal sections (posterior hippocampus/mid-thalamus level) per animal (sham, n = 3; HI, n = 3). The investigator who performed the differential count was blinded to the experimental conditions. In each region of interest, O4 positive cells were counted in eight randomly chosen fields of view with a 40× objective equipped with a counting grid (0.0625 mm^2^) using a Nikon Eclipse E400 microscope (Nikon, Amsterdam, The Netherlands).

Differential counts of MPO positive cells in the brain were performed to assess the localization of these cells in relation to the cerebral vasculature. Numbers of intravascular, perivascular and interstitial cells were counted in the hippocampus, periventricular white matter and subcortical white matter. Six coronal sections per animal (sham, n = 3; HI, n = 3) were studied at the posterior hippocampus/mid-thalamus level. In each section, cells were counted in eight fields of view (focused on the cerebral vasculature) per region of interest (hippocampus, periventricular white matter and subcortical white matter) with a 20x objective equipped with a counting grid (0.25 mm^2^) using a Nikon Eclipse E400 microscope (Nikon, Amsterdam, The Netherlands).

Since in the MPO analysis the fields of view were not randomly chosen, but focused on the cerebral vasculature, the numbers of cells were expressed as cells per field of view (FOV).

All images of immunohistochemical staining in the brain (IBA-1, O4, MBP, MPO) presented here were obtained with an Olympus AX-70 microscope (Olympus, Tokyo, Japan) equipped with a digital camera.

### White blood cell counts

Automated white blood cell counts were performed in heparinized arterial blood on experimental days −3, 0, 1, 3, 5 and 7 using a Sysmex XE-5000 hematology analyzer (Sysmex, Etten-Leur, The Netherlands).

### Immunohistochemistry spleen

Spleens were removed immediately following sacrifice and subsequently weighed. Tissue blocks (5 × 5 mm^2^) were snap frozen in liquid nitrogen. Frozen spleen sections (4 μm) were stained for cleaved caspase-3 (Asp175, #9661S, Cell Signaling Technology, Boston, MA, USA) for detection of apoptosis, CD3 (DAKO A0452, DAKO, Glostrup, Denmark) for detection of T-cells and MPO (DAKO A0398, DAKO) for detection of neutrophils.

Endogenous peroxidase was inactivated by incubation with 0.3% H_2_O_2_ that was dissolved in methanol. Antigen specific binding was prevented by incubating the slides for 30 minutes with 5% bovine serum albumin (BSA). Slides were incubated overnight at 4°C with the diluted primary antibody (cleaved caspase-3 1:200, CD3 1:200, MPO 1:500) followed by incubation with the appropriate secondary biotin labeled antibody. Immunostaining was enhanced with Vectastain ABC peroxidase Elite kit (PK-6200, Vector Laboratories) followed by a NiDAB staining. Sections were counterstained with 0.1% Nuclear Fast Red washed, dehydrated and coverslipped. The number of caspase-3 positive cells in the spleen were counted in twenty (to accommodate heterogenic distribution) fields of view per animal (sham, n = 6; HI, n = 6) with a 20× objective equipped with a counting grid (0.25 mm^2^) using a Nikon Eclipse E400 microscope. The number of caspase-3 positive cells was expressed in cells/mm^2^.

For the analysis of CD3 and MPO immunoreactivity (IR), digital images of spleen sections were acquired at 100× magnification using a Leica DM200 microscope equipped with a Leica DFC295 digital camera (Leica Microsystems) and Leica Application Suite (LAS) software (Leica LAS V 3.7, Leica Microsystems). Areal fraction of CD3 and MPO IR was determined in five sections per animal (sham, n = 6; HI, n = 6) with a standard threshold to determine positive staining using Leica Qwin software (Leica Qwin Pro V 3.5.1, Leica).

### Flow cytometry

At the end of the experiment (day 7), the spleen was immediately harvested after sacrifice. Single-cell splenocyte suspensions were obtained by dissociating freshly sampled spleen tissues in gentleMACS™ C-tubes (MiltEnyi, Leiden, The Netherlands) filled with Gibco® Iscove’s Modified Dulbecco’s Medium (IMDM) (Life Technologies, Bleiswijk, The Netherlands) using the gentleMACS™ Dissociator (MiltEnyi). Subsequently, the cell suspensions were passed through a 70 μm cell strainer (BD Biosciences, Erembodegem-Aalst, Belgium). Splenocytes were stored in nitrogen in freezing medium containing IMDM medium with 10% heat-inactivated fetal calf serum and 10% dimethylsulfoxide (DMSO).

To study the cellular composition of the spleen 7 days after global HI, 200,000 splenocytes per animal (sham, n = 8; HI, n = 8) were stained for detection of lymphocytes (mouse anti sheep CD45-biotin; AbDSerotec, Düsseldorf, Germany/streptavidin-Horizon V450; BD Biosciences), neutrophils (mouse anti-bovine CD11b-Fluorescein isothiocyanate (−FITC); AbDSerotec, Düsseldorf, Germany), T-helper cells (mouse anti sheep CD4-AlexaFluor® 647 (−A647); AbDSerotec), cytotoxic T-cells (mouse anti sheep CD8-R-phycoerythrin (−PE); AbDSerotec) and viability (7-Aminoactinomycin D (7-AAD); BD Biosciences) according to the manufacturer’s protocol. Stained cells were acquired on a FACS Canto II flow cytometer (BD Biosciences) equipped with FACS Diva software (BD Biosciences). The number of CD11b, CD4 and CD8 positive splenocytes were determined as a percentage of living CD45-positive lymphocytes. The expression of CD11b, CD4 and CD8 on living CD45-positive lymphocytes was analyzed using the mean fluorescent intensity (MFI).

### Statistics

Summary statistics of animal characteristics (gestational age at UCO, body weight) are shown as means with 95% confidence intervals (CI). For analysis of O4, MPO and activated caspase-3 parameters, cell counts in each section’s region of interest were first averaged per field of view (n = 8 for O4 and MPO; n = 20 for activated caspase-3). Groups’ comparisons (sham *vs*. HI) with respect to all outcome parameters were drawn either with independent *t*-tests, or with random intercept models in case of repeated measurements per animal (e.g. different sections per brain). Variables, whose distributions were positively skewed, were log-transformed previous to statistical testing. To facilitate interpretation, averages on the log scale were back transformed to the original scale (antilog) and are presented as geometric means and corresponding 95% CIs.

Average (additive) differences on log transformed data become ‘multiplicative’ on the original scale. Thus, the displayed geometric means for the sham and HI groups should be compared in relative terms, not as difference in averages (mean sham minus mean HI), but rather as a ratio of the sham geometric mean with respect to the HI geometric mean (mean sham divided by mean HI). The interpretation of the geometric means ratio is provided for example for areal fraction (%) IBA-1 immunoreactivity in the subcortical white matter (see Results section).

For analysis of the EEG parameter (IBI length), log transformation also preceded parametric inferences regarding groups’ comparisons and temporal dynamics of mean IBI length before and after UCO (or sham). To accommodate both the interrupted nature of the experimental follow-up, with UCO happening on day 0 of the experiment, as well as the correlation among longitudinal measurements of individual fetuses, a piecewise mixed regression model was fitted [27]. This mixed model approach allowed additionally for heterogeneity of groups’ variances to be accounted for. In the model, time (pre and post UCO, measured in days), group (sham *vs.* HI), and a dummy for pre and post UCO times (pre-post), were the fixed effects factors. Fetuses (subjects) were the random factor. The addition of random effects was meant to model individual variability relative to the group’s average. Variables selection was carried out via the top-down procedure based on likelihood ratio (LR) tests for fixed effects and tests for the covariance structure.

Statistical analysis was performed with PASW Statistics 18 (SPSS Inc., Chicago, IL, USA).

## Results

### Animal characteristics

Fetal body weight did not differ between the sham and HI group; sham mean: 1,782 g (1571; 1993) *versus* HI mean: 1,742 g (1482; 2002), *P* = 0.677. There was no significant difference in gestational age at the time of UCO between the sham and HI groups; sham mean: 105.6 days (104.6; 106.5) *versus* HI mean: 105.5 days (104.7; 106.3), *P* = 0.717.

### Fetal vital parameters

Fetal vital parameters and blood gases during UCO are depicted in Figure 1. After an initial compensatory rise, mean arterial blood pressure gradually declined from 40 mmHg to 10 mmHg at the end of 25 minutes UCO (Figure 1A). Mean fetal heart rate rapidly fell after initiation of UCO from around 200 beats per minute (bpm) at baseline to below 100 bpm at the end of UCO (Figure 1B). All vital parameters normalized within 30 minutes of reperfusion time.

Blood gas data (Figure 1C-E) indicate that average pH dropped from 7.4 at baseline to 6.8 at the end of UCO. Mean partial oxygen pressure decreased from 20 mmHg at baseline to values below 5 mmHg at the end of UCO. Mean partial carbon dioxide pressure increased from baseline levels of 40 mmHg to values around 90 mmHg at the end of UCO. Upon reperfusion hypoxemia and hypercapnia resolved within minutes. Normalization of pH values occurred after 60 to 90 minutes (data not shown).

### Brain atrophy

**Figure 3:**
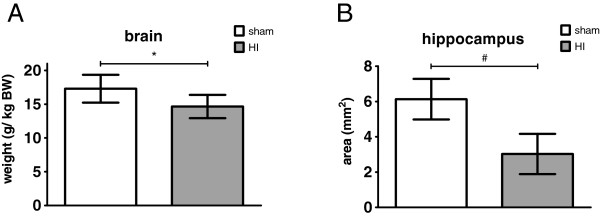
**Global HI induced atrophy of the brain and the hippocampus.** This is indicated by a significant reduction of brain weight (g/kg BW) (**A**) and hippocampus area (mm^2^) (**B**). (**A**) Means ± 95% CI are depicted. (**B**) Geometric means ± 95% CI are depicted. *P ≤0.05, # *P* ≤0.01, ‡ *P* ≤0.001, NS, non-significant; HI = hypoxia-ischemia.

Brain weight, corrected for body weight (BW), and hippocampal area, were determined to study HI-induced brain atrophy. Average brain weight (g/kg BW) was significantly decreased in animals exposed to HI compared with sham (Figure 3A); sham mean: 17.3 g/kg BW (95% CI 15.2; 19.4) *versus* HI mean: 14.6 g/kg BW (95% CI 12.9; 16.4), *P* = 0.037.

Atrophy of the hippocampus was assessed since there is clinical [3-5] and experimental [19,28] evidence that this brain region is affected in preterm HIE. Mean area (mm^2^) of the hippocampus was significantly reduced in animals exposed to HI compared with sham (Figure 3B); sham mean: 6.1 mm^2^ (95% CI 5.0; 7.3) *versus* HI mean: 3.0 mm^2^ (95% CI 1.9; 4.2), *P* = 0.002.

In hippocampal sub-regions the analysis of the areas (mm^2^) showed significant atrophy of the *cornu ammonis* (CA)1-2; sham mean: 1.7 mm^2^ (95% CI 1.3; 2.0) *versus* HI mean: 0.7 mm^2^ (95% CI 0.4; 1.0), *P* = 0.001 and CA3; sham mean: 0.7 mm^2^ (95% CI 0.6; 0.9) *versus* HI mean: 0.4 mm^2^ (95% CI 0.2; 0.6), *P* = 0.013.

Mean area of the dentate gyrus (DG) was not significantly affected by HI; sham mean: 1.2 mm^2^ (95% CI 1.0; 1.4) *versus* HI mean: 0.7 mm^2^ (95% CI 0.6; 1.1), *P* = 0.060.

### Microglial activation and proliferation

**Figure 4:**
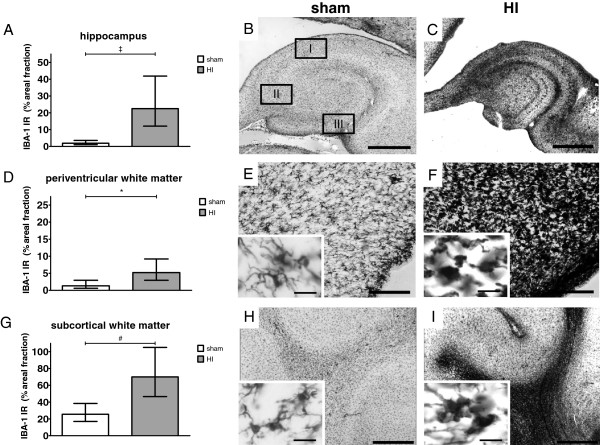
**Global HI induced activation and proliferation of microglia in the hippocampus, PVWM and SCWM.** Microglial proliferation and activation was shown by significantly increased areal fraction (%) of IBA-1 immunoreactivity (IR) and by loss of processes and amoeboid morphology, respectively. (**A**) Global HI significantly increased areal fraction (%) of IBA-1 IR in the hippocampus; (**B**) Sham, hippocampus; resting microglia, also depicting the analyzed hippocampal sub-regions (see Figure 5); I = *cornu ammonis* (CA)1-2, II = CA3, III = dentate gyrus (DG); (**C**) HI, hippocampus; profound microglial proliferation and activation (scale bar = 500 μm); (**D**) Global HI significantly increased areal fraction (%) of IBA-1 IR in the PVWM; (**E**) Sham, PVWM; resting microglia (scale bar = 200 μm, scale bar insert = 25 μm); (**F**) HI, PVWM; proliferation and activation of microglia (scale bar = 200 μm, scale bar insert = 25 μm); (**G**) Global HI significantly increased areal fraction (%) of IBA-1 IR in the SCWM; (**H**) Sham, SCWM; resting microglia (scale bar = 200 μm, scale bar insert = 25 μm); (**I**) HI, SCWM proliferation and activation of microglia (scale bar = 200 μm, scale bar insert = 25 μm). (**A**, **D**, **G**) Geometric means ± 95% CI are depicted. *P ≤0.05, # *P* ≤0.01, ‡ *P* ≤0.001, NS, non-significant. HI = hypoxia-ischemia.

Microglia (IBA-1) were studied to determine the local inflammatory response in the brain. Areal fraction (%) of IBA-1 immunoreactivity (IR) was studied in the SCWM, PVWM and hippocampus (Figure 2A). IBA-1 IR was significantly increased in the SCWM of animals exposed to HI compared with sham (Figure 4D-F); sham geometric mean: 25.6% (95% CI 17.0; 38.4) *versus* HI geometric mean: 70.0% (95% CI 46.6; 105.1), *P* = 0.003. Thus, the ratio of the two geometric means, HI with respect to sham is 70.0/25.6 = 2.73. The corresponding interpretation is that the geometric mean of the areal fraction in the HI group is 2.73 higher (173% increase) than the geometric mean of the sham group.

In the PVWM the areal fraction (%) of IBA-1 IR was significantly increased in animals exposed to HI compared with sham (Figure 4A-C); sham geometric mean: 1.3% (95% CI 0.6; 3.0) *versus* HI geometric mean: 5.2% (95% CI 2.9; 9.2), *P* = 0.013.

The areal fraction (%) of IBA-1 IR in the hippocampus was significantly increased in animals exposed to HI compared with sham (Figure 4G-I); sham geometric mean: 1.9% (95% CI 1.0; 3.6) *versus* HI geometric mean: 22.5% (95% CI 12.1; 41.8), *P* <0.001.

**Figure 5:**
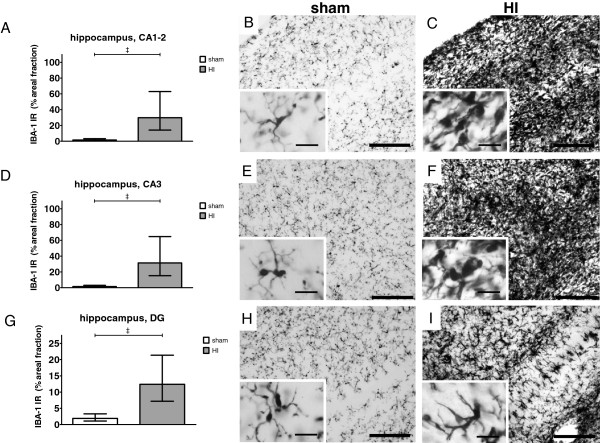
**Global HI induced significant proliferation and activation of microglia in the *cornu ammonis *(CA)1-2, CA3 and dentate gyrus (DG), sub-regions of the hippocampus.** (**A**) Global HI significantly increased areal fraction (%) of IBA-1 immunoreactivity (IR) in CA1-2; (**B**) Sham, CA1-2; resting microglia; (**C**) HI, CA1-2; profound microglial proliferation and activation; (**D**) Global HI significantly increased areal fraction (%) of IBA-1 IR in CA3; (**E**) Sham, CA3; resting microglia; (**F**) HI, CA3; profound proliferation and activation of microglia; (**G**) Global HI significantly increased areal fraction (%) of IBA-1 IR in DG; (**H**) Sham DG; resting microglia; (**I**) HI, DG; proliferation and activation of microglia. Scale bar images = 200 μm, scale bar inserts = 25 μm. (A, D, G) Geometric means ± 95% CI are depicted. *P ≤0.05, # *P* ≤0.01, ‡ *P* ≤0.001, NS, non-significant. HI = hypoxia-ischemia.

Analysis of hippocampal sub-region CA1-2 showed significantly increased IBA-1 IR in animals exposed to HI compared to sham (Figure 5A-C); sham geometric mean 1.5% (95% CI 0.7; 3.2) *versus* HI geometric mean 29.8% (95% CI 14.1; 63.1), *P* <0.001. IBA-1 IR was also significantly increased in CA3 (Figure 5D-F); sham geometric mean 1.4% (95% CI 0.7; 3.0) *versus* HI geometric mean 31.4% (95% CI 15.2; 64.8), *P* <0.001 and DG (Figure 5G-I); sham geometric mean 1.3% (95% CI 0.6; 3.0) *versus* HI geometric mean 5.2% (95% CI 2.9; 9.2), *P* <0.001.

Microglia in sham animals exhibited a quiescent state characterized by extensively branched thin processes (inserts Figure 4E and H, and Figure 5B, E and H). In contrast, in HI-exposed animals, microglia with thick cell bodies and retracted processes were observed indicating an activated state (inserts Figure 4F and I, and Figure 5C, F and I).

### Region-specific pre-oligodendrocyte vulnerability

**Figure 6:**
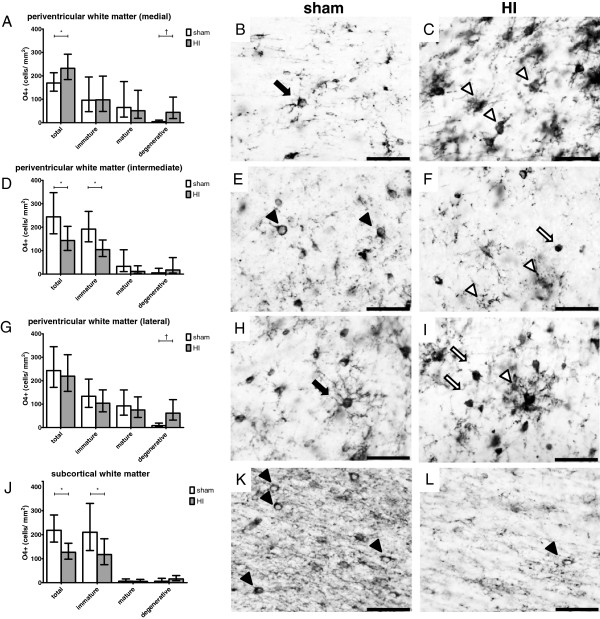
**Global HI induced region-specific vulnerability of O4-positive pre-oligodendrocytes (preOLs; late oligodendrocyte progenitors and immature oligodendrocytes).** Regions of interest are depicted in Figure 2. (**A**) Global HI significantly increased the density of total and degenerative preOLs in the medial periventricular white matter (PVWM). (**B**) Sham, medial PVWM; preOL with mature phenotype (black arrow). (**C**) HI, medial PVWM; preOLs with degenerative phenotype (white arrowheads). (**D**) Global HI significantly decreased density of total and immature preOLs in the intermediate PVWM. (**E**) Sham, intermediate PVWM; preOLs with immature phenotype (black arrow heads). (**F**) HI, intermediate PVWM; preOLs with degenerative phenotype (white arrow heads) and apoptotic preOL (white arrow). (**G**) Global HI significantly increased the density of degenerative preOLs in the lateral PVWM, density of total preOLs was unchanged. (**H**) Sham, lateral PVWM; preOL with mature phenotype (black arrow). (**I**) HI, lateral PVWM; preOL with degenerative (white arrow head) and apoptotic (white arrow) phenotype. (**J**) Global HI significantly reduced the density of total and immature preOLs subcortical white matter (SCWM). (**K**) Sham, SCWM; preOLs with immature phenotype (black arrow heads). (**L**) HI, SCWM; reduced density of preOLs with immature phenotype (black arrow heads) and disturbance of O4-positive myelin sheath organization. Scale bar all images = 50 μm. (**A**, **D**, **G**, **J**) Geometric means ± 95% CI are depicted. *P ≤0.05, # *P* ≤0.01, ‡ *P* ≤0.001, NS, non-significant. HI = hypoxia-ischemia.

Differential counts of O4-positive preOLs were performed in the medial, intermediate and lateral PVWM and in the SCWM (Figure 2B). Analysis of the O4 staining showed that in sham animals the medial and lateral PVWM were predominantly populated by O4-positive cells with a mature phenotype (Figure 6A-B and G-H). In contrast, O4-positive cells in the SCWM and intermediate PVWM were predominantly of immature phenotype (Figure 6D-E and J-K). Following global HI all regions showed an increase in preOLs with degenerative morphology (Figure 6A, D, G and J) which reached statistical significance in the regions which were populated by mature preOLs in sham conditions (medial and lateral PVWM). In the medial PVWM, the areal density of O4-positive cells with degenerative phenotype significantly increased in animals exposed to HI compared to sham (Figure 6A-C); sham geometric mean 4.6 cells/mm^2^ (95% CI 1.9; 11.3) *versus* HI geometric mean 44.2 cells/mm^2^ (95% CI 17.8; 109.4), *P* = 0.009. In the lateral PVWM, the areal density of O4-positive cells with degenerative morphology similarly increased following HI (Figure 6G-I); sham geometric mean 8.3 cells/mm^2^ (95% CI 3.6; 18.8) *versus* HI geometric mean 61.6 cells/mm^2^ (95% CI 31.9; 119.1).

Following global HI, the total number of preOLs significantly decreased in those regions which were populated by immature preOLs in sham conditions (intermediate PVWM and SCWM). In the intermediate PVWM, the areal density of total O4-positive cells significantly decreased following global HI (Figure 6D-F); sham geometric mean 244.2 cells/mm^2^ (95% CI 171.7; 347.2) *versus* HI geometric mean 143.2 cells/mm^2^ (95% CI 100.7; 203.6), *P* = 0.041. Loss of total preOLs in the intermediate PVWM was mainly attributable to loss of O4-positive cells with immature phenotype (Figure 6D-F); sham geometric mean 191.9 cells/mm^2^ (95% CI 137.7; 267.5) *versus* HI geometric mean 104.3 cells/mm^2^ (95% CI 74.8; 145.3), *P* = 0.023.

The areal density of total O4-positive cells in the SCWM significantly decreased in HI-exposed animals compared to sham (Figure 6J-L); sham geometric mean: 218.3 cells/mm^2^ (95% CI 169.2; 282.0) *versus* HI geometric mean: 126.8 cells/mm^2^ (95% CI 98.0; 164.0), *P* = 0.014. Loss of total preOLs in the SCWM was mainly attributable to loss of O4-positive cells with immature phenotype (Figure 6J-L); sham geometric mean: 210.2 cells/mm^2^ (95% CI 133.9; 330.3) *versus* HI geometric mean: 117.2 cells/mm^2^ (95% CI 75.0; 183.1), *P* = 0.014. Moreover, in the SCWM a clear disturbance of O4-positive myelin sheath organization was observed following HI (Figure 6L). Remarkably, in the medial PVWM the total number of preOLs significantly increased (Figure 6A); sham geometric mean: 169.4 cells/mm^2^ (95% CI 134.4; 213.4) *versus* HI geometric mean: 232.1 cells/mm^2^ (95% CI 184.2; 292.4), *P* = 0.038.

### MBP

**Figure 7:**
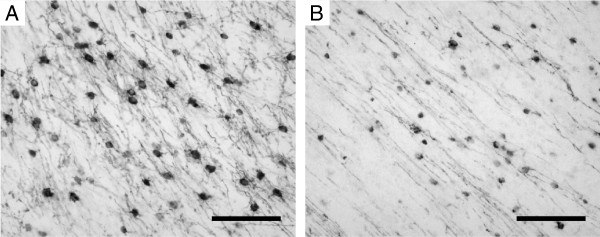
**Global HI induced white matter injury.** This was shown by marked loss of myelin basic protein (MBP) positive myelin sheaths and myelin-producing cells (mature oligodendrocytes) in the subcortical white matter. (Region of interest indicated in Figure 2).

Brain sections were stained for myelin basic protein (MBP) to detect white matter injury following HI. In sham animals abundant MBP-positive myelin sheaths and myelin-producing cells (mature oligodendrocytes) were observed in the subcortical white matter (Figure 7). Following global HI a marked reduction of both myelin sheaths and mature oligodendrocytes was observed (Figure 7). In the PVWM (regions 1, 2 and 3 in Figure 2) no MBP immunoreactivity was detected in both sham and HI animals (data not shown) indicating that these white matter regions were not myelinated at this developmental stage of the preterm brain.

### Cerebral neutrophil invasion

**Figure 8:**
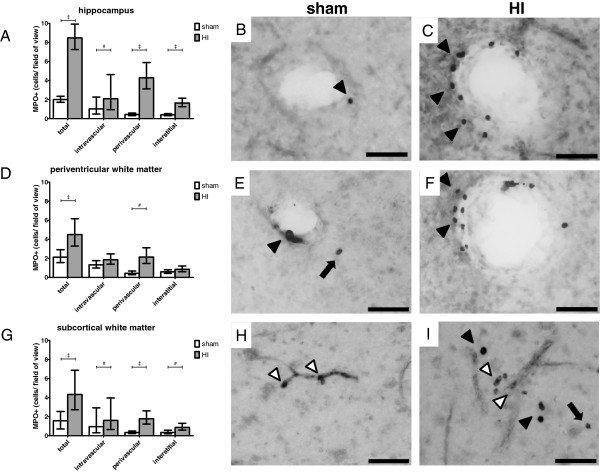
**Global HI induced significant invasion of MPO-positive cells (neutrophils) into the hippocampus, PVWM and SCWM.** Invading neutrophils were predominantly localized in the perivascular zone. (**A**) Global HI caused a significant increase in the total number of MPO-positive cells in the hippocampus, mainly attributable to a profound increase in the number of perivascular MPO-positive cells; (**B**) Sham, hippocampus; perivascular MPO-positive cell (black arrow head); (**C**) HI, hippocampus; profound increase in the number of perivascular MPO-positive cells (black arrow heads); (**D**) Global HI caused a significant increase in the total number of MPO-positive cells in the PVWM, attributable to a significant increase in the number of perivascular MPO-positive cells; (**E**) Sham, PVWM; perivascular (black arrow head) and interstitial (black arrow) MPO-positive cells; (**F**) HI, PVWM; marked increase in perivascular MPO-positive cells (black arrow heads); (**G**) Global HI caused a significant increase in the total number of MPO-positive cells in the SCWM, attributable to a significant increase in the number of intravascular, perivascular and interstitial MPO-positive cells; (**H**) Sham, SCWM; intravascular MPO-positive cells (white arrow heads); (**I**) HI, SCWM; intravascular (white arrow heads), perivascular (black arrow heads) and interstitial (black arrow) MPO-positive cells. Scale bar all images = 50 μm. (A, D, G) Geometric means ± 95% CI are depicted. *P ≤0.05, # *P* ≤0.01, ‡ *P* ≤0.001, NS, non-significant. HI = hypoxia-ischemia.

Brain sections were stained for MPO to detect neutrophils that invaded the brain following HI. Although microglia can also produce MPO, neutrophils and microglia can be easily distinguished by intensity of staining, localization and morphology [29-31]. Following global HI the total number of MPO-positive cells inside or adjacent to the cerebral vasculature significantly increased in the hippocampus (Figure 8A-C); sham geometric mean: 2.0 cells/field of view (FOV) (95% CI 1.7; 2.4) *versus* HI geometric mean: 8.5 cells/FOV (95% CI 2.7; 9.9), *P* <0.001. Similarly, the total number of MPO-positive cells significantly increased in the PVWM (Figure 8D-F); sham geometric mean: 2.1 cells/FOV (95% CI 1.6; 2.9) *versus* HI geometric mean: 4.5 cells/FOV (95% CI 3.3; 6.2), *P* = 0.002; and in the SCWM (Figure 7G-I), sham geometric mean: 1.6 cells/FOV (95% CI 0.7; 2.5) *versus* HI geometric mean: 4.3 cells/FOV (95% CI 2.7; 6.9), *P* <0.001.

All analyzed regions showed an increase of intravascular MPO-positive cells following global HI, which reached significance in the hippocampus (Figure 8A); sham geometric mean: 1.0 cells/FOV (95% CI 0.5; 2.3) *versus* HI geometric mean: 2.1 cells/FOV (95% CI 0.9; 4.6), *P* = 0.002; and in the SCWM (Figure 8G), sham geometric mean: 1.0 cells/FOV (95% CI 0.3; 2.9) *versus* HI geometric mean: 1.6 cells/FOV (95% CI 0.7; 4.0), *P* = 0.009.

The number of perivascular MPO-positive cells was significantly increased in the hippocampus of HI-exposed animals (Figure 8A-C); sham geometric mean: 0.4 cells/FOV (95% CI 0.3; 0.6) *versus* HI geometric mean: 4.3 cells/FOV (95% CI 3.1; 5.9), *P* <0.001. Likewise, the number of perivascular MPO-positive cells was significantly increased in the PVWM of HI-exposed animals (Figure 8D-F), sham geometric mean: 0.4 cells/FOV (95% CI 0.3; 0.7) *versus* HI geometric mean: 2.1 cells/FOV (95% CI 1.5; 3.1), *P* <0.001; and in the HI-exposed SCWM (Figure 8G-I), sham geometric mean: 0.3 cells/FOV (95% CI 0.2; 0.5) *versus* HI geometric mean: 1.8 cells/FOV (95% CI 1.2; 2.6), *P* <0.001.

All analyzed regions showed an increase of interstitial MPO-positive cells following global HI, which reached significance in the hippocampus (Figure 8A), sham geometric mean: 0.4 cells/FOV (95% CI 0.3; 0.5) *versus* HI geometric mean: 1.6 cells/FOV (95% CI 1.3; 2.1), *P* <0.001; and in the SCWM (Figure 8G), sham geometric mean: 0.3 cells/FOV (95% CI 0.2; 0.6) *versus* HI geometric mean: 0.9 cells/FOV (95% CI 0.6; 1.3), *P* = 0.007.

MPO-positive cells in the brain sections studied were large round-shaped cells with lobular intracellular structures situated in and around the cerebral vasculature (Figure 8B-C, E-F and H-I). These morphological features and their localization indicate that these MPO-positive cells were neutrophils. MPO-positive cells lacking neutrophil morphology were rarely detected (data not shown).

### White blood cell mobilization

**Figure 9:**
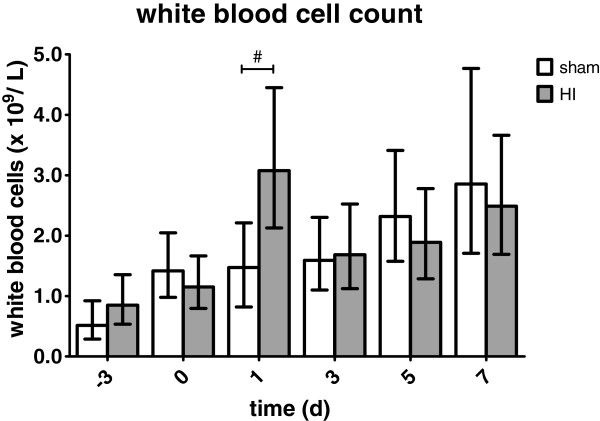
**Global HI induced significant white blood cell mobilization in the first 24 hours following umbilical cord occlusion (UCO).** White blood cell counts gradually increased during the study period. Geometric means ± 95% CI are depicted. *P ≤0.05, # *P* ≤0.01, ‡ *P* ≤0.001, NS, non-significant. HI = hypoxia-ischemia.

One day post-UCO a significant increase in white blood cell count was observed (Figure 9); sham geometric mean: 1.5 × 10^9^ cells/L (95% 0.8; 2.2) *versus* HI geometric mean: 3.1 × 10^9^ cells/L (95% CI 2.1; 4.5), *P* = 0.009.

Furthermore, white blood cell counts showed a gradual increase in the number of circulating white blood cells during the study period in both sham and HI animals (Figure 9). When geometric means were compared to sham day −3 (0.5 × 10^9^ cells/L (95% CI 0.3; 0.9)), white blood cell counts of sham animals significantly increased at day 0 (1.4 × 10^9^ cells/L (95% CI 1.0; 2.0); *P* = 0.027), day 1 (1.5 × 10^9^ cells/L (95% CI 0.8; 2,2); *P* = 0.024), day 3 (1.6 × 10^9^ cells/L (95% CI 1.1; 2.3); *P* = 0.008), day 5 (2.3 × 10^9^ cells/L (95% CI 1.6; 3.4); *P* <0.001) and day 7 (2.9 × 10^9^ cells/L (95% CI 1.7; 4.8); *P* <0.001). In HI animals geometric mean white blood cell counts were significantly elevated compared to HI day −3 (0.9 × 10^9^ cells/L (95% CI 0.5; 1.4)) at day 1 (3.1 × 10^9^ cells/L (95% CI 2.1; 4.5); *P* <0.001) and day 7 (2.5 × 10^9^ cells/L (95% CI 1.3; 3.7); *P* = 0.002).

Flow cytometry analysis of whole blood showed that the percentage of living (7-AAD negative) CD45-positive lymphocytes expressing CD11b (neutrophils), CD4 (helper T-cells) or CD8 (cytotoxic T-cells), did not differ between sham and HI groups in the circulation on day 1 (data not shown). This indicated that global HI induced non-preferential mobilization of immune cells 24 hours following global HI.

### Splenic involution

**Figure 10:**
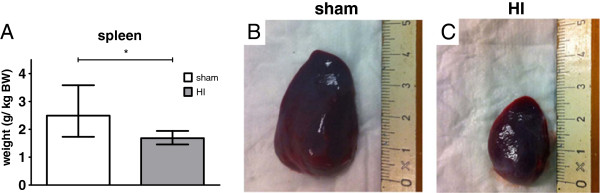
**Global HI resulted in splenic involution.** Global HI significantly reduced spleen weight (g/kg BW) (**A**) and size (**B**, **C**). (B) Sham, spleen. (**C**) HI, spleen. (A) Geometric means ± 95% CI are depicted. *P ≤0.05, # *P* ≤0.01, ‡ *P* ≤0.001, NS, non-significant. HI = hypoxia-ischemia.

Seven days after global HI, splenic weight was analyzed as an indication of activation of the peripheral immune system. Spleen weight, corrected for fetal BW, was significantly decreased in fetuses exposed to HI (Figure 10A); sham geometric mean 2.5 g/kg BW (95% CI 1.7; 3.6) *versus* HI geometric mean 1.7 g/kg BW (95% CI 1.5; 1.9), *P* = 0.033. Consistently, spleen size was markedly reduced following HI (Figure 10B).

**Figure 11:**
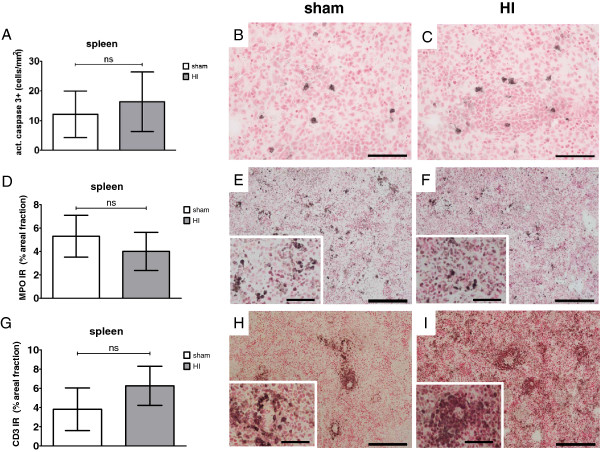
**(A) Splenic apoptosis was not affected by global HI as indicated by unchanged numbers (cells/mm**^**2**^**) of activated caspase-3 positive cells in the spleen assessed seven days after umbilical cord occlusion (UCO); (B) Sham, spleen; activated caspase-3; (C) HI, spleen; activated caspase-3; (D) Global HI did not change areal fraction (%) of MPO immunoreactivity in the spleen seven days after UCO; (E) Sham, spleen; MPO; (F) HI, spleen; MPO; (G) Global HI did not change areal fraction (%) of CD3 immunoreactivity in the spleen seven days after UCO; (H) Sham, spleen; CD3; (I) HI, spleen; CD3. Scale bar activated caspase-3 images = 50 μm.** Scale bar CD3 and MPO images = 200 μm, scale bar inserts = 50 μm. (A, D, G) Means ± 95% CI are depicted. *P ≤0.05, # *P* ≤0.01, ‡ *P* ≤0.001, NS, non-significant. HI = hypoxia-ischemia

To assess whether splenic involution was caused by increased apoptotic cell death, spleen sections were stained for activated caspase-3. The number (cells/mm^2^) of activated caspase-3 positive cells in the spleen did not differ between sham and HI groups (Figure 11A-C); sham mean: 12.2 cells/mm^2^ (95% CI 4.4; 20.0) *versus* HI mean: 16.4 cells/mm^2^ (95% CI 6.4; 26.4), *P* = 0.391.

### Splenic cellular composition

Immunohistochemical staining of spleen sections for neutrophils showed that areal fraction (%) of MPO immunoreactivity (IR) in the spleen did not differ between sham and HI animals after a reperfusion time of 7 days (Figure 11D-F); sham mean: 5.3% (95% CI 3.5; 7.1) *versus* HI mean: 4.0% (95% CI 2.4; 5.6), *P* = 0.254. Immunohistochemical staining of spleen sections for T-cells showed that areal fraction (%) of CD3 IR in the spleen did not differ between sham and HI animals after a reperfusion time of 7 days (Figure 11D-F); sham mean: 3.8% (95% CI 1.6; 6.0) *versus* HI mean: 6.3% (95% CI 4.2; 8.3), *P* = 0.100.

**Figure 12:**
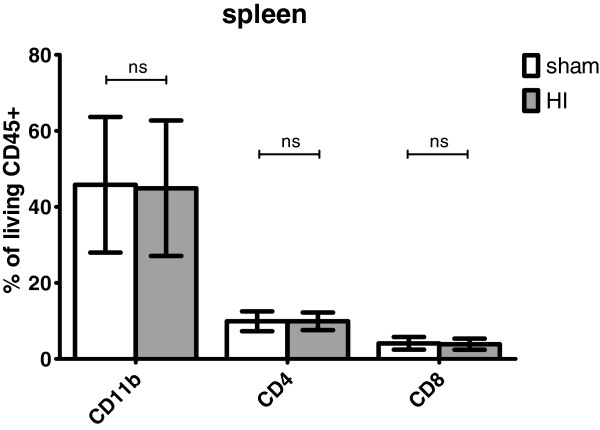
**Global HI did not affect the number of splenocytes expressing CD11b, CD4 or CD8 seven days after umbilical cord occlusion (UCO).** Means ± 95% CI are depicted. *P ≤0.05, # *P* ≤0.01, ‡ *P* ≤0.001, NS, non-significant. HI = hypoxia-ischemia.

Similarly, no differences in splenic cell populations were found between sham and HI animals using flow cytometry on splenocytes. The percentage of living (7-AAD negative) CD45-positive splenocytes expressing CD11b (neutrophils) did not differ between sham and HI groups (Figure 12); sham mean: 45.8% (95% CI 28.0; 63.7) *versus* HI mean: 44.9% (95% CI 27.1; 62.7), *P* = 0.936. The percentage of living CD45-positive splenocytes expressing CD4 (helper T-cells) was not changed 7 days following HI (Figure 12); sham mean: 9.9% (95% CI 7.3; 12.5) *versus* HI mean: 9.9% (95% CI 7.6; 12.2), *P* = 0.996. The percentage of living splenocytes in the spleen expressing CD8 (cytotoxic T-cells) remained also unchanged 7 days following HI (Figure 12); sham mean; 4.1% (95% CI 2.4; 5.8) *versus* HI mean: 3.9% (95% CI 2.4; 5.3), *P* = 0.825. In line with these data, expression levels of CD11b, CD4 and CD8 on living CD45+ splenocytes, as measured with mean fluorescence intensity (MFI), were not changed by global HI after a reperfusion period of seven days (data not shown).

### EEG suppression

**Figure 13:**
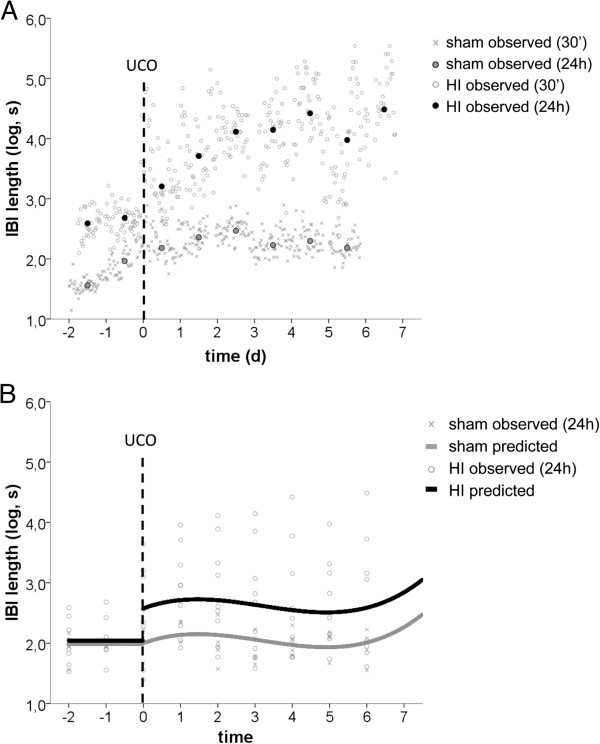
**Global HI resulted in prolonged profound suppression of preterm brain function.** (**A**) Observed mean IBI length (log scale) per 24 hours of two animals, one sham and one HI, superimposed on observed mean IBI length per 30 minutes; (**B**) Values predicted by the mixed linear model are superimposed on observed IBI length per 24 hours of all animals. IBI = interburst interval length, log = natural logarithm, s = seconds, UCO = umbilical cord occlusion, 30’ = 30 minutes, 24h = 24 hours, d = day.

The fetal EEG was continuously recorded in HI-exposed (n = 8) and sham (n = 8) fetuses during the complete study period. EEG analysis was performed from two days before UCO until the end of the experiment after a reperfusion period of seven days. Interburst interval (IBI) length was assessed to determine suppression of brain function following HI. EEG suppression was indicated by prolonged IBI length. Figure 13A displays the observed IBI length values over time (background; grey) of two animals, one sham fetus and one HI fetus, averaged over 30 minutes. Time point ‘0’ indicates day of UCO. Note the distinct difference in IBI length between the HI *versus* sham fetuses after occlusion, with more prolonged length and larger fluctuations for the former. Superimposed on the 30 minutes data in Figure 13A are also the IBI length values averaged over 24 hours, for the same two animals (foreground; full circles). For model simplicity, the piecewise regression model was fitted on the 24 hours data.

Figure 13B displays the IBI length temporal dynamics as estimated by the final regression model. Its fixed and random effect parameters are displayed in Tables 1 and 2. The predicted lines (according to the model) are superimposed on observed 24 hour values (all animals). Note the greater IBI length variability in the HI fetuses induced by the occlusion. This more variable responsiveness was captured in the model by the significant random slope variance for in the HI group (Tables 1 and 2).

**Table 1:** Estimated fixed and random effect parameters of the piecewise (mixed) regression model

**Parameters**	**(95% CI)**
(Fixed and random effects)	n = 16
Intercept	2.60 (2.19, 3.00) ‡
Group (Sham)	−7.14 (−1.39, -0.03) *
Dummy pre-post UCO (pre-time)	−0.63 (−0.95, -0.32) ‡
Group*pre-post dummy	0.65 (0.27, 1.02) ‡
Pre-UCO time	−0.01 (−0.20, 0.18) NS
Post-UCO time	0.29 (0.12, 0.46) ‡
Post-UCO time (2)	−0.12 (−4.84, 1.73) ‡
Post-UCO time (3)	0.01 (0.00, 0.02) ‡
Residual variance	0.03 (0.02, 0.05) ‡
Random intercept variance, pre-UCO time	0.29 (0.12, 0.68) *
Random slope variance, pre-UCO time	0.03 (0.01,0.10) *
Random slope variance, post-UCO time HI	0.007 (0.002, 0.20)*

Estimated fixed and random effect parameters of the piecewise (mixed) regression model (Outcome variable: mean IBI length, on the log scale. Covariance structure: diagonal). The fixed effect regression coefficients β for the continuous time variables indicate average changes in mean IBI length per day. For nominal variables, β represent average changes in the highlighted category (in brackets) with respect to the reference group (omitted). *P ≤0.05, # *P* ≤0.01, ‡ *P* ≤0.001, NS, non-significant.

**Table 2:** Interpretation of model parameters

**Group (Sham)**	**Captures IBI length differences in means between SHAM and HI averaged over all time points**
Dummy pre-post UCO (pre-time)	Captures the (vertical) shift of mean IBI values, induced by the interrupted treatment (UCO)
Group*pre-post dummy	Captures the differential shift of mean IBI length values immediately after UCO - significant only for the HI group
Pre-UCO time	Captures a linear increase of mean IBI length over time before UCO Note: Non-significant
Post-UCO time	All 3 time associated parameters capture curve-linear changes of mean IBI lengths over time after UCO
Post-UCO time (2)	
Post-UCO time (3)	
Residual variance	Captures within subjects variability (noise)
Random intercept variance, pre-UCO time	Captures between subject variability at the outset
Random slope variance, pre-UCO time	Captures between subject variability with respect to linear IBI changes over time before UCO (each animal has its own slope)
Random slope variance, post-UCO time HI	Captures heterogeneity of between subject variability with respect to linear IBI changes over time after UCO; larger variable responsiveness for the HI group

There is a clear upwards shift (increase) in the average IBI length values (log scale) for the HI group compared to sham after UCO (significant interaction between pre-post dummy and group variables, capturing the average change in IBI length level for the HI group immediately after UCO). Mean IBI length after UCO remained higher throughout the measured time span (one week) for the HI group. With respect to temporal changes, it is noteworthy to mention that before UCO the averaged values remain stable over time (pre-UCO time variable did not reach statistical significance), contrary to post-UCO time. After UCO, IBI length seemed to change in a curve-linear pattern for both groups (significant quadratic and cubic post-time parameters in the model). In summary, IBI length was significantly increased following UCO and remained higher in the HI group during the seven day reperfusion time, indicating prolonged suppression of preterm brain function following global HI.

## Discussion

In this study we showed that global HI caused profound inflammation of the preterm ovine brain which was paralleled by mobilization of the peripheral innate immune system. These inflammatory changes were associated with suppressed brain function, brain atrophy, region-specific vulnerability of preOLs and hypomyelination, which are known to correlate with white matter disease, the clinical hallmark of preterm HIE [13].

Microglial proliferation following global HI was demonstrated by immunohistochemical staining of IBA-1, a specific marker for microglia under normal and neuroinflammatory conditions [24-26,32,33]. Moreover, we showed morphological transformation of IBA-1 positive microglia from ramified into amoeboid state indicating activation of these cells following global HI in preterm sheep [34,35]. These microglial changes, as seen after global ischemia in preterm lambs, are in line with the microglial response after focal ischemia of the adult brain which typically occurs within 24 hours after the insult [36-38].

Mobilization of the peripheral innate immune system in our model was demonstrated by a non-preferential recruitment of immune cells into the circulation within 24 hours following global HI as well as a marked influx of neutrophils into the HI-exposed preterm hippocampus and white matter seven days after the global HI insult. This invasion of neutrophils, which is an important histopathological finding in cerebral ischemia, was primarily localized in the perivascular zone [39-41]. The influx of neutrophils, which typically occurs as a second hit within 48 to 72 hours after cerebral ischemia, is considered to further aggravate acute inflammation of the brain that was initiated by immediate cell death and microglial activation by enhanced free radical attack [36-38].

A permeable blood brain barrier (BBB) is a prerequisite for the influx of neutrophils such as seen following global HI [41,42]. Clinical [43] and experimental [44-46] evidence showed disruption of the BBB in perinatal HI. The influx of neutrophils also indicates that the immature immune system is capable of responding to an inflammatory stimulus induced by global HI. This concept is in line with recent literature challenging the dogma that the preterm immune system is naive [47-49]. However, the fact that neutrophils were predominantly observed in the perivascular zone following HI, and to a lesser extent in the interstitium, indicated that the capacity of fetal neutrophils to transmigrate was immature [48].

We postulate that the cerebral neutrophils, as seen in our model, were derived from the spleen, since this organ is considered to be the predominant source of invading neutrophils following focal ischemia of the brain [18,50,51]. This concept is further supported by work of Ajmo *et al.* who showed in a rat model of focal cerebral ischemia that splenectomy reduced neutrophil influx and microglial activation, ultimately diminishing ischemic brain injury [17]. In line with this hypothesis, we observed that neutrophil invasion into the preterm brain following global HI was associated with marked splenic involution. Since splenic apoptosis was not affected by global HI, we suggest that involution of the spleen was caused by mobilization of neutrophils and other immune cells rather than HI-induced splenic cell death. However, 7 days after global HI we did not detect changed immune cell composition in the spleen. The latter is in line with a neonatal mouse study of Winerdal *et al.* which showed an altered splenic cellular response between 24 and 72 hours after HI, subsequently neutrophil invasion into the brain peaked 7 days after ischemia [52]. Together these findings suggest that splenic cellular changes in our model occurred within hours after the global HI-insult. This shows an important limitation of the current study in which temporal dynamics of the splenic cellular immune response were not studied. Notably, HI-induced involution of the spleen may have clinical postnatal consequences since splenic involution has been associated with an increased risk of postnatal infectious complications such as early onset sepsis [21,53]. Splenectomy in our model of global HI is required to confirm the role of the spleen as a source of neutrophils.

The cerebral and systemic inflammatory changes observed in our study were accompanied by prolonged suppression of preterm brain function. The persistent reduction in brain activity as observed in our study is in line with previous studies in comparable ovine models of global HI [22,54]. Clinical evidence showed that persistent suppression of EEG activity is associated with poor outcome, indicating the severity of the HI-insult applied in this study [55,56]. Furthermore, global HI induced severe brain atrophy in our study which has previously been associated with neuronal injury [57,58].

Our findings showed that HI-induced cerebral inflammation was paralleled by region-specific preOL vulnerability. In the SCWM, loss of morphologically immature O4-positive preOLs was associated with hypomyelination, which is in concordance with previous results in a similar model of HI sheep [22,54]. In the PVWM, we observed loss of morphologically immature preOLs as well as increased numbers of morphologically mature preOLs following global HI. These findings suggested that the preOL response following global HI varied from degeneration to proliferation and depended on the region and the morphological maturity of preOLs. This concept is supported by recent studies suggesting that myelination failure of the preterm brain following HI may be caused by a combination of preOL degeneration, regeneration and arrested maturation [59-61]. The observed white matter injury and functional impairments following global HI are typical findings in preterm infants with HIE underlining the translational character of the preterm sheep model.

## Conclusions

This study provides evidence that cerebral inflammation and mobilization of the peripheral innate immune system are paralleled by injury and functional loss of the preterm brain following global HI. We postulate that the spleen plays a key role in preterm HIE by providing immune effector cells to the circulation and subsequently to the injured brain. Further studies should focus on the interplay between preterm brain inflammation and the activation of the peripheral immune system following global HI. Better understanding of the involvement of cerebral and systemic inflammation in the course of global HI will contribute to the development of future therapeutic interventions in preterm HIE.

## Abbreviations

BSA: Bovine serum albumin; CI: Confidence interval; ECG: Electrocardiogram; EEG: Electroencephalogram; HI: Hypoxia-ischemia or hypoxic-ischemic; HIE: Hypoxic-ischemic encephalopathy; IBA-1: Ionized calcium binding adaptor molecule 1; IBI: Interburst interval; Log: Natural logarithm; MBP: Myelin basic protein; MPAQ: Maastricht-Programmable AcQuisition system; MPO: Myeloperoxidase; NiDAB: Nickel sulfate-diaminobenzidine; PreOLs: Pre-oligodendrocytes; PVWM: Periventricular white matter; SCWM: Subcortical white matter; SD: Standard deviation; UCO: Umbilical cord occlusion; VSI: Virtual slide imaging

## Competing interests

The authors of this manuscript declare that there are no actual or potential conflicts of interest. The authors affirm that there are no financial, personal or other relationships with other people or organizations that have inappropriately influenced or biased their research.

## Authors’ contribution

RJ designed and performed the animal experiments, analyzed the data and wrote the manuscript under the supervision of TW and BK. VLP performed and supervised the statistical analysis. AZ, WJ and PA performed and interpreted the EEG analysis. DO, JJC and MS performed animal experiments. SM and EK performed immunohistochemistry. JPC designed custom-made software for the analysis of immunohistochemistry. JV, WG, HV, HS, AG, TW and BK significantly contributed to conception, design, data interpretation and editing of the manuscript. All authors read and approved the final manuscript.
